# A Putative Case of Methotrexate-Related Lymphoma: Clinical Course and PET/CT Findings

**DOI:** 10.1155/2009/469343

**Published:** 2009-12-23

**Authors:** Rachel C. Jankowitz, James Ganon, Todd Blodgett, Christine Garcia, Samuel Jacobs

**Affiliations:** ^1^Department of Oncology, University of Pittsburgh, Pittsburgh, PA 15213, USA; ^2^Division of Hematology/Oncology, Magee Womens Hospital, University of Pittsburgh Medical Center, 300 Halket Street, Pittsburgh, PA 15213, USA; ^3^Department of Radiology, University of Pittsburgh, Pittsburgh, PA 15213, USA; ^4^Department of Pathology, University of Pittsburgh, Pittsburgh, PA 15213, USA

## Abstract

Patients with autoimmune conditions develop lymphoproliferative disorders (LPDs) at a higher frequency than normal both in association with and independent of Methotrexate (MTX). We describe a case of MTX-associated lymphoma in a patient with psoriasis on long-standing MTX. The case is notable for the initial tumor burden, the dramatic disappearance of the PET-CT findings on discontinuation of MTX, and the subsequent early regrowth of disease. Our case report is illustrative of an MTX-related NHL in an autoimmune patient. *Conclusion*. Withdrawal of MTX in a patient with lymphoma is reasonable before initiating chemotherapy, but observation for early regrowth of disease is necessary.

Patients with autoimmune and inflammatory disorders develop lymphoproliferative disorders (LPDs) at a higher frequency than the normal population [1]. Immune dysregulation, both hyperimmunity and/or immunodeficiency, may underlie such LPD [2]. Rheumatoid arthritis (RA) patients have an approximately twofold increase in incidence of lymphoma [2], and patients with severe psoriasis have a modestly increased incidence of lymphoma: 7.9/100,000 per year [3]. 

Methotrexate (MTX) is an antimetabolite and immunosuppressant used in the treatment of autoimmune conditions. Its mechanism of predisposing patients to LPD is unclear [4], as patients with autoimmune conditions develop LPD both in association with and independent of MTX administration. The association between MTX and lymphoma was first reported in a patient on weekly oral pulse-methotrexate in 1993 [5], and now numerous case studies have reported similar findings [6–16]. It has been suggested that the disease state of RA, not specific therapy, underlies the development of lymphoma in RA patients [2, 17]. Cases of MTX-related lymphomas are often extranodal in origin, are often EBV-associated [7], and can respond to withdrawal of immunosuppression [18]. 

We describe a case of MTX-associated LPD in a patient with longstanding, severe psoriasis. The remarkable features of this case include the tumor burden at presentation, the rapid and dramatic disappearance of the PET-CT findings on discontinuation of MTX, and the subsequent early re-growth of disease.

The patient is a 70-year-old gentleman with a history of Hepatitis B exposure, chronic obstructive pulmonary disease (COPD), coronary artery disease, hypertension, and psoriasis treated with MTX for two years. He had been taking a 20 mg weekly dose of MTX and had never been treated with any other immunosuppressive agents. Three weeks prior to presenting at our clinic, he stopped MTX due to deteriorating health. For two months, he had poor appetite, early satiety, and alternating diarrhea and constipation. He had intermittent crampy abdominal pain, a 20-pound weight loss, fatigue, and occasional night sweats without fever or pruritus. Physical exam revealed an elderly gentleman who appeared depressed and fatigued. He was afebrile. His blood pressure was 104/58, his heart rate was 62, and his respiratory rate was 14. His skin showed no active psoriatic lesions, and he had no palpable peripheral lymphadenopathy. Head and neck exam revealed no jaundice, and a clear oral pharynx. Heart and lung exams were normal. He had a protuberant abdomen with a tender palpable liver that extended below the xiphoid. There was no ascites or splenomegaly. 

WBC count was 8.9 × 10^9^/L with normal differential, hemoglobin was 12.3 g/dL, and platelet count was 347 × 10^9^/L. LDH was 667 IU/L (nl. range 313–618 IU/L), alkaline phosphatase was 198 IU/L (nl. range 38–126 IU/L); and SGOT and ALT were normal. CEA was <0.5 ng/mL, and AFP was 3 ng/mL. Colonoscopy was negative.

Initial PET/CT scan (Figure 1) showed widespread FDG-avid lymphadenopathy in the neck, axillae, upper abdomen, retroperitoneum, pelvis, and inguinal regions. Extensive bony abnormalities were present in the thorax, spine, and pelvis. Multiple FDG-avid lesions were in the liver; the largest measured 8.9 cm.

Liver biopsy by Fine Needle Aspiration (FNA) 1 week after presentation showed atypical spindly cells in a background of lymphoid cells with severe crush artifact and was nondiagnostic for tumor. Repeat core liver biopsy (Figure 2) was also negative for malignant cells, showing a lymphoid infiltrate that lacked cytologic atypia. Immunostains showed predominantly CD3+ T cells with very rare CD20+ small B cells; EBV-encoded RNA in situ hybridization (EBER ISH) was negative (Figures 2(a)–2(d)). Molecular studies for T cell receptor gene rearrangement by PCR analysis were negative.

Bone marrow biopsy 3 weeks after presentation showed no evidence of neoplasm and normal cytogenetics. FNA of an inguinal lymph node the next day was also nondiagnostic and devoid of lymphoid tissue. 

PET/CT scan that was repeated at one month (Figure 1) and off MTX for 7 weeks showed dramatic decrease in all areas of lymphadenopathy, with complete resolution of abnormal FDG-uptake in his liver, bone, and lymph nodes. The patient felt much better, had gained 6 pounds of weight, and denied B-symptoms or abdominal pain. 

Unfortunately, repeating PET/CT three months later (Figure 1) showed interval progression of disease with new onset of conglomerate lymphadenopathy in multiple lymph node regions as well as a new enhancing lesion near the head of the pancreas, a soft tissue nodule inferior to the right hepatic lobe, and new-onset hypermetabolic lesions involving the right and left iliac bones.

A biopsy of the right axillary lymph node showed reactive changes as well as a focal cluster of abnormal follicles lacking polarization with numerous large cells. Immunostains showed that the large cells were BCL6+ BCL2−, and confirmed intact, although focally effaced, CD21+ follicular dendritic meshworks (Figures 2(e)–2(h)). EBER ISH was negative. Cytogenetic studies revealed a clonal population with multiple karyotypic abnormalities, including involvement of the immunoglobulin heavy chain gene (14q32), and supported involvement by B cell lymphoma. Molecular studies were negative for clonal T and B cell rearrangements.

Laparoscopic-guided retroperitoneal biopsy of the juxta-gastric lymph node was performed and established the diagnosis of B cell lymphoma, large cell type. Histologic sections demonstrated a diffuse infiltrate comprised of large CD20 positive B lymphoid cells in a background of histiocytes and small lymphocytes, scattered BCL6 + large lymphoid cells, and EBER ISH was negative (Figures 2(i)–2(l)). The proliferation index was 30%, as highlighted by Ki-67 immunostaining. In the diffuse areas, underlying CD21+ follicular dendritic cell meshworks were not identified. However follicular dendritic meshworks were identified in a few BCL6+ BCL2+ nodules of large cells, reminiscent of the prior right axillary lymph node specimen. Although no metaphases were obtained for classical cytogenetic studies, FISH studies performed on paraffin-embedded tissue on both nodal specimens showed similar results and were positive for an Immunoglobulin heavy locus (IGH) rearrangement and negative for rearrangements of the *MYC, BCL6, BCL2, *and *BCL3 *loci. The findings suggested a similar pathologic process in both specimens, with likely focal follicular colonization in the prior right axillary lymph node specimen by large cell lymphoma in light of the pathologic features from the juxta-gastric lymph node. 

 Treatment was initiated with standard doses of Cyclophosphamide, Adriamycin, Vincristine, Prednisone, and Rituximab (CHOP-R).

Patients with autoimmune and chronic inflammatory disorders develop lymphoma at a higher frequency than the normal population [7]. It is unclear why such relatively hyperimmune patients are predisposed to lymphoma. Chronic antigen stimulation by exogenous or endogenous antigens such as viruses or bacteria, in the setting of chronic inflammation and decreased immune surveillance, is hypothesized to underlie the transformation of B-cells to malignant clones [2]. While such antigenic stimulation drives B-cell immunoglobulin gene rearrangement, constitutive oncogenic expression may occur, with resultant uncontrolled clonal proliferation of B-cells [2]. 

EBV normally causes asymptomatic primary infection of B-cells, but it does occasionally fuel a malignant transformation process, particularly in germinal-center B-cells [19]. Cases of MTX-related LPDs are often EBV-associated [7] and may respond to simple withdrawal of immunosuppression [18] suggesting that restoration of normal immunity may suppress EBV-related lymphomas. Feng studied the effect of MTX and other immunosuppressants on EBV replication in vitro and found that it activated the release of infectious EBV from latently infected cell lines and that patients treated with MTX had higher blood-levels of EBV than those on other immunosuppressives [20].

Despite the association of MTX-related LPDs and EBV-positivity, our EBV-negative patient showed dramatic reduction in disease burden with resolution of abnormal FDG-uptake on PET/CT after withdrawal of MTX as can be seen in Figure 1. Similarly, in a Japanese study of 76 RA patients with LPD [6], those treated with MTX developed LPDs in a shorter period of time than those who were not on the drug. After withdrawal of MTX, spontaneous regression of masses occurred in 11 of the 48 cases and continued for 8 to 64 months. Six of the 11 cases responding to MTX withdrawal were EBV-positive, but 5 were EBV-negative like our patient. Three out of 5 cases that had recurrence of LPD after initial response to MTX withdrawal were EBV-negative. The higher recurrence rate in the EBV-negative LPD group may have been related to their underlying autoimmune disease and/or an inability to control chronic inflammation.

Unfortunately, our patient also experienced a short disease-free interval of only 2 months in response to MTX withdrawal. Biopsy at the time of recurrence was diagnostic of a malignant lymphoma, large B cell type, which was EBV-negative. We can only speculate that at presentation, the patient may have had an EBV-positive B-cell lymphoma with complete lysis of the EBV-positive clones as the MTX was withdrawn. Alternatively, our patient may represent a case of EBV-negative MTX-related lymphoma with rapid but short-lived regression upon withdrawal of MTX. 

Our report is illustrative of an MTX-related NHL in a patient with a chronic inflammatory state treated with immunosuppressive therapy. It was striking in this case how difficult it was to make a tissue diagnosis despite the markedly abnormal PET/CT. There was very rapid improvement in his scans after MTX withdrawal, only to be followed by regrowth of disease within 2 months. Based on this case and others in literature, we suggest withdrawal of MTX in any patient diagnosed or suspected of having lymphoma before initiation of systemic chemotherapy, even in cases with florid disease. However, close initial observation for early regrowth of disease even in responding patients is warranted.

## Figures and Tables

**Figure 1:**
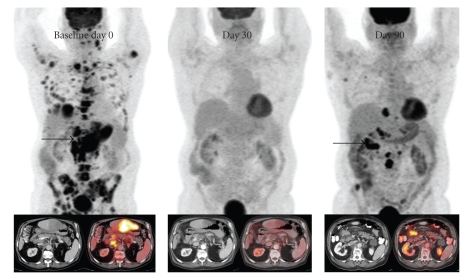
Imaging Test Results: images from 3 different PET/CT scans are shown above. Images from the patient′s initial study are on the left and show extensive abnormal areas of FDG activity on the coronal PET image and correlative CT and fused PET/CT images (inset left) of the large lesion in the liver. A second scan performed 30 days after the baseline scan shows complete resolution of the abnormal FDG activity (middle group of images) in all lesions after discontinuation of the patient′s methotrexate. No other treatment was initiated. Note persistent low attenuation lesion in the liver (inset middle). Image on the right from a repeat scan 90 days later shows multiple new lesions, most of which were not even present on the initial scan.

**Figure 2:**
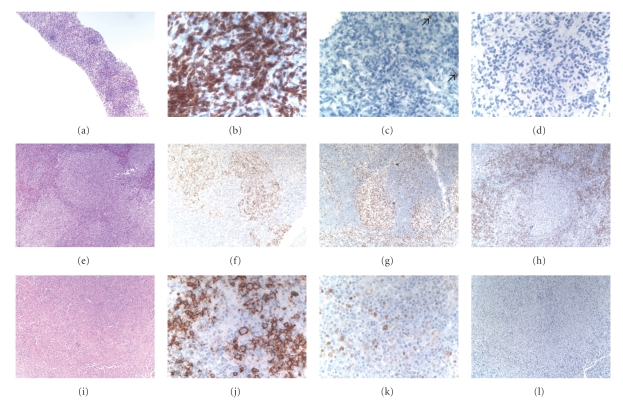
Pathology: (a) H and E section of liver core biopsy with dense infiltrate of small lymphocytes, original magnification 100X. (b) CD3 immunostain showing predominance of small T cells, original magnification 400X. (c) CD20 immunostain showing rare positive small B cells (arrows), original magnification 400X. (d) EBV encoded RNA in-situ hybridization (EBER), original magnification 400X. (e) H and E section of right axillary lymph node showing cluster of abnormal follicles, original magnification 100X. (f) CD21 immunostain showing intact but focally effaced follicular dendritic meshworks, original magnification 100X. (g) BCL6 immunostain showing many positive cells in follicles, original magnification 100X. (h) BCL2 immunostain with numerous negative cells in follicles, original magnification 100X. (i) H and E section of juxta-gastric lymph node demonstrating diffuse infiltrate of histiocytes and small and large lymphoid cells, original magnification 100X. (j) CD20 immunostain highlighting large lymphoid cells within infiltrate, original magnification 400X. (k) BCL6 immunostain showing scattered positive cells, including large lymphoid cells, original magnification 400X. (l) EBV encoded RNA in-situ hybridization (EBER), original magnification 100X.
